# Steady-State NTPase Activity of Dengue Virus NS3: Number of Catalytic Sites, Nucleotide Specificity and Activation by ssRNA

**DOI:** 10.1371/journal.pone.0058508

**Published:** 2013-03-19

**Authors:** J. Jeremías Incicco, Leopoldo G. Gebhard, Rodolfo M. González-Lebrero, Andrea V. Gamarnik, Sergio B. Kaufman

**Affiliations:** 1 Instituto de Química y Fisicoquímica Biológicas and Departamento de Química Biológica, Facultad de Farmacia y Bioquímica, Universidad de Buenos Aires, Ciudad Autónoma de Buenos Aires, Argentina; 2 Fundación Instituto Leloir-CONICET, Ciudad Autónoma de Buenos Aires, Argentina; University of Nebraska, United States of America

## Abstract

Dengue virus nonstructural protein 3 (NS3) unwinds double stranded RNA driven by the free energy derived from the hydrolysis of nucleoside triphosphates. This paper presents the first systematic and quantitative characterization of the steady-state NTPase activity of DENV NS3 and their interaction with ssRNA. Substrate curves for ATP, GTP, CTP and UTP were obtained, and the specificity order for these nucleotides - evaluated as the ratio (*k_cat_*/*K_M_*)- was GTP
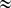
ATP
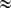
CTP 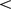 UTP, which showed that NS3 have poor ability to discriminate between different NTPs. Competition experiments between the four substrates indicated that all of them are hydrolyzed in one and the same catalytic site of the enzyme. The effect of ssRNA on the ATPase activity of NS3 was studied using poly(A) and poly(C). Both RNA molecules produced a 10 fold increase in the turnover rate constant (*k_cat_*) and a 100 fold decrease in the apparent affinity (*K_M_*) for ATP. When the ratio [RNA bases]/[NS3] was between 0 and 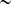
20 the ATPase activity was inhibited by increasing both poly(A) and poly(C). Using the theory of binding of large ligands (NS3) to a one-dimensional homogeneous lattice of infinite length (RNA) we tested the hypothesis that inhibition is the result of crowding of NS3 molecules along the RNA lattices. Finally, we discuss why this hypothesis is consistent with the idea that the ATPase catalytic cycle is tightly coupled to the movement of NS3 helicase along the RNA.

## Introduction

The dengue virus (DENV) is a member of the family *Flaviviridae*, which includes other major public health concerns such as yellow fever virus, hepatitis C virus and West Nile virus [1]. Dengue virus exists in four distinct serotypes; all of them are mosquito-borne and cause dengue fever and dengue hemorrhagic fever/dengue shock syndrome. Found in tropical and subtropical regions of the world, DENV causes an estimated of 50 –100 million infections annually and places over 3 billion people at risk for infection [2], [3].

The genome of Flaviviruses is a positive-sense single stranded RNA molecule of about 11,000 nucleotides bases constituted by a single open reading frame flanked by conserved and highly structured 5′ and 3′ untranslated regions [4], [5]. The entire viral genome is translated as a single polyprotein which is subsequently cleaved by viral and cellular proteases into functional entities. At least 10 viral proteins are produced: three structural proteins (capsid, premembrane, and envelope proteins) and seven nonstructural proteins (NS1, NS2A, NS2B, NS3, NS4A, NS4B and NS5 proteins) [4], [6], [7].

Dengue virus non-structural protein 3 (NS3) is a multifunctional protein of 618 amino acids built of two functional domains: an N-terminal region (residues 1–167) that forms a two-component serine protease domain together with residues of NS2B and a C-terminal region (residues 171–618) endowed with RNA-helicase, nucleoside 5′-triphosphatase (NTPase), 5′ -terminal RNA triphosphatase (RTPase), as well as RNA annealing activities [8]–[11]. These two functional domains may act independently of each other *in vitro* and recombinant NS3 lacking the protease domain retains NTPase and helicase activities [9], [11].

The crystal structure of DENV NS3 NTPase/helicase has been determined at 0.24 nm resolution [12]. Its tertiary structure is characterized by three domains, each of about 130–150 amino acids. The first two domains (domains I and II) are RecA-like domains, which constitute the core of most ATP-driven molecular motors [13], [14], and host seven characteristic sequence motifs of superfamily 2 DExH helicases [15], [16]. In particular, the conserved motifs I (GAGKTRR) and II (DEAH), also known as Walker A or ‘P-loop’ and Walker B motifs respectively, interact with nucleotides and Mg^2+^ [17], [18]. The cleft formed between domain III and the other two presents numerous basic residues and is wide enough to accommodate a single-stranded nucleic acid substrate but not a duplex [18].

NS3 is an RNA helicase, these proteins are an ubiquitous class of enzymes that participate in virtually all processes of the RNA metabolism [19]. Many viruses encode proteins with *in vitro* helicase activity, but their specific roles in viral replication are still unclear. A common feature shared by these motor proteins is their capability to catalyze the hydrolysis of nucleoside triphosphates (NTPs), which provides the driving force for the rearrangement of the RNA structures.

In contrast to the amount of structural information available for DENV NS3, the characterization of its functional properties is rather incomplete. It is known that DENV NS3 helicase binds preferentially to single-stranded RNA, while low affinity was observed for single or double-stranded DNA (dsDNA) molecules. In addition, it requires a single stranded 3′ overhang to unwind dsRNA substrates and thus it is held that NS3 translocates in the 3′ to 5′ direction [20].

It has been shown that DENV NS3 catalyzes the hydrolysis of nucleotides ATP, CTP, GTP and UTP, that Mg^2+^ as well as Mn^2+^ are essential activators of the NTPase activity and that this activity is stimulated by ssRNA [8], [9], [21]. Regarding the steady-state kinetics of NTP hydrolysis catalyzed by NS3 only the substrate curves for ATP can be found in the literature and little is known about the dependence of NTPase activity on RNA concentration. On the other hand, it was observed that the above mentioned four nucleotides drive the unwinding activity of NS3 [11] but the steady-state kinetic parameters and the specificity of NS3 toward these substrates were not yet established.

In this paper we report quantitative studies on the steady-state kinetics of NTP hydrolysis catalyzed by DENV NS3. We performed substrate curves for the nucleotides ATP, GTP, CTP and UTP and established the specificity order among them according to the (*k_cat_*/*K_M_*) values. Additionally, we demonstrated that the four nucleotides are hydrolyzed in one and the same catalytic site of the enzyme.

When the ATPase activity of NS3 was studied as a function of the concentration of long polyribonucleotides poly(A) and poly(C) it was found that these RNA molecules can act as activators or inhibitors of the ATPase activity depending on the ratio [RNA]/[NS3]. Taking into account the treatment developed by McGhee *et. al.* [22] for the binding of large ligands (NS3) to a homogeneous one-dimensional lattice of infinite length (RNA) we formulated a kinetic model that was able to describe adequately the effect of long ssRNA on the ATPase activity of DENV NS3.

## Results

### NTPase activity in the absence of RNA

#### Substrate curves and nucleotide specificity by NS3

**Figure 1:**
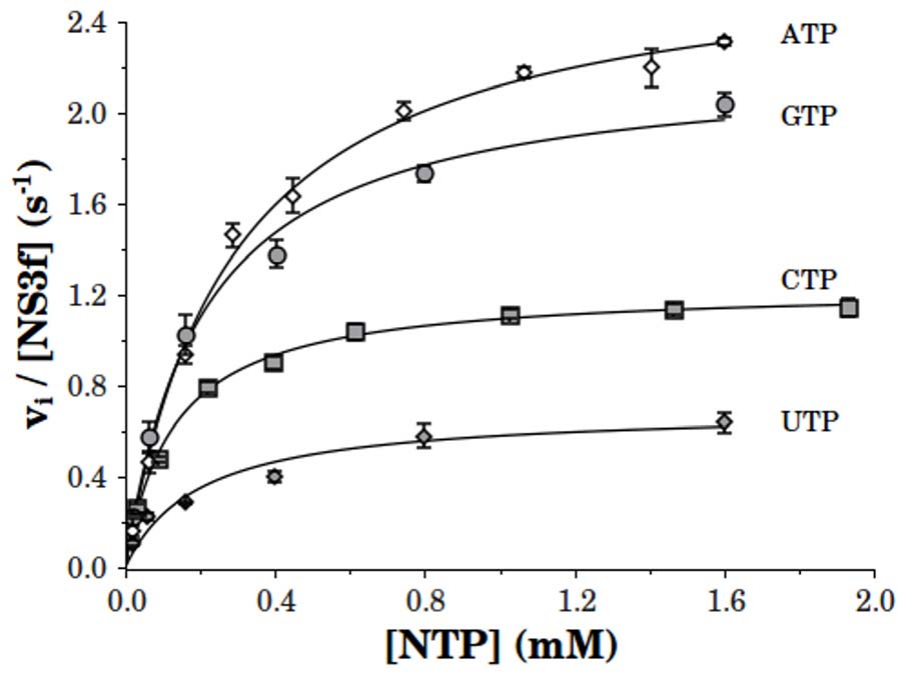
Steady-state NTPase activity of NS3f as a function of substrate concentration. Initial rates of NTP hydrolysis were obtained for ATP (empty diamond), GTP (filled circle), CTP (filled square) and UTP (filled diamond).The experiments were performed using 50 nM NS3f in a reaction media containing 100 mM KCl and all other reagents as indicated in Materials and Methods. Continuous lines are plots of hyperbolic functions whose parameter values (*k_cat_* and *K_M_*) were obtained by non-linear regression analysis and are shown in Table1.

We began the characterization of the kinetics properties of the NS3 helicase studying the hydrolysis of nucleotides ATP, CTP, GTP and UTP catalyzed by NS3 under steady-state conditions. It can be seen (Figure 1) that NS3 catalyzes the hydrolysis of the 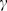-phosphate group of the four nucleotides tested. The substrate curves followed single hyperbolic functions with *k_cat_* and *K_M_* values shown in Table 1.

**Table 1:** Parameters of the steady-state NTPase activity of NS3f in the absence of RNA.

NTP	K_M_ (mM)		k_cat_ (s^-1^)		k_cat_/K_M_ (10^3^M^-1^s^-1^)	
ATP	0.28±0.02		2.71±0.06		9.6±0.6	
GTP	0.20±0.03		2.22±0.09		11±1	
CTP	0.13±0.01		1.23±0.01		9.6±0.5	
UTP	0.20±0.06		0.69±0.07		3.5±0.9	

*k_cat_*, *K_M_* and *k_cat_*/*K_M_* values (SE) are for the substrate curves shown in Figure 1. These parameters corresponds to a single hyperbolic function.

Specificity for each nucleotide was analyzed according to the *k_cat_*/*K_M_* values. These specificity constants (*k_cat_*/*K_M_*) are used to determine the ratio of reaction rates for competing substrates as described in the following equation:
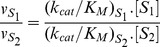
(1)where 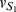 and 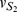 are the rates of the enzyme catalyzed reaction for the transformation of substrate *S_1_* and *S_2_*, respectively, when both substrates are present together in the reaction media at the concentrations [*S_1_*] and [*S_2_*]. Thus, the ratio between *k_cat_*/*K_M_* values for two given substrates is a measurement of the ability of the enzyme to discriminate in favor of one substrate in the presence of another one.

The results shown in Table 1 indicate that the substrate specificity order for DENV NS3 was: GTP
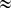
ATP
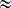
CTP 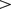 UTP.

#### The nucleotides ATP, CTP, GTP and UTP compete for the same catalytic site

We investigated whether the four nucleotides are hydrolyzed in the same catalytic site of NS3. To address this issue we made use of the fact that if two different substrates yield the same product (as in the present case, where orthophosphate is produced from the four nucleotides) it is possible to distinguish between a kinetic model of competition for the same site from others of multiple catalytic sites by measuring the steady-state velocity of product formation in mixtures of both substrates [23], [24]. This is because the analytical expression for the steady-state velocity as a function of the concentration of both substrates is different for these kinetic models.

**Figure 2:**
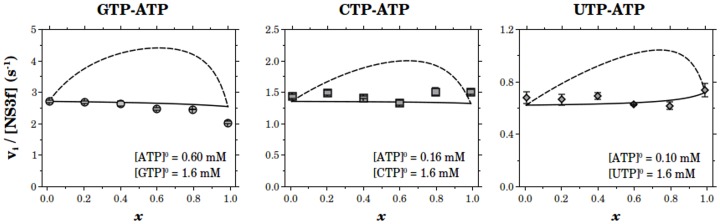
Competition plots for ATP and a second nucleotide. Initial rates of phosphate release (*vi*) were measured in reaction mixtures containing two nucleotides at a time. The value of abscissa *x* is the proportion of [ATP] relative to the reference concentration [ATP]0 (see text). All experiments were performed using 51 nM NS3f and 100 mM KCl while other components were as indicated in Materials and Methods. *k_cat_* and *K_M_* values obtained from substrate curves in Figure 1 were used to simulate the prediction of the single-active-site model (continuous line) and the two-independent-active-sites model (dashed line) according to the following equations: 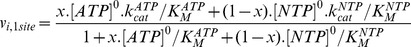 and 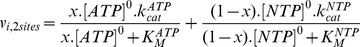. The former model shows the better correspondence with these results.

According to the procedure described by Chevillard *et. al.* [24], we carried out a series of experiments in which ATP and a second NTP were present in the same reaction media and the initial rate of total orthophosphate release was measured. We prepared a series of reaction mixtures containing ATP and a second NTP at concentrations given by:
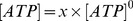
(2)

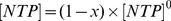
(3)where *x* is an arbitrary factor that takes values between 0 and 1 and [*ATP*]^0^ and [*NTP*]^0^ were chosen in such a way that at these concentrations each substrate yields the same reaction rate (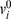) in the absence of the other. For each reaction mixture the rate of total phosphate release was measured and the results were plotted as a function of *x* (Figure 2). If both substrates react in the same catalytic site the reaction rate would be independent of *x*, and thus a horizontal straight line would be obtained. This behavior is different from that arising from other simple kinetic models. For instance, completely independent reactions generate a curve with a maximum whereas antagonistic reactions generate a curve with a minimum. This method [24] can be extended to the more general case where the values of 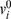 at the concentrations [*ATP*]^0^ and [*NTP*]^0^are not exactly equal. It can be demonstrated that when both substrates react in the same catalytic site the resulting plot would be a monotonous hyperbola, whereas the other two possibilities would give rise to non monotonous dependences on *x*.

Figure 2 shows the results of the measurement of the NTPase activity in the presence of ATP and GTP, CTP or UTP. It can be seen that in the three cases the rate of phosphate release varied almost linearly with *x*. Along with the experimental data, the simulations of the expected results for two different kinetic models are shown: (1) one catalytic site for all nucleotides (continuous line) or (2) different and independent catalytic sites for each nucleotide (dashed line). As will be further discussed later (see Discussion) these results strongly supports the hypothesis that ATP, GTP, CTP and UTP react in a same catalytic site on the NS3 helicase.

### ATPase activity in the presence of RNA

In what follows, all experiments were performed upon the isolated helicase domain of NS3, NS3h, instead of the full length construct. The principal reason for this is that NS3f co-purifies with lower molecular weight SDS-PAGE bands whereas these are absent in NS3h preparation. In addition NS3h is purified with greater yields than the full length counterpart. Such properties make the NS3h preparation the most suitable for studies on the binding and stoichiometry of the NS3-RNA interaction.

On the other hand, it was shown in a previous work that NS3h and NS3f present equal functional properties such as NTPase and helicase activities and annealing of RNA [11]. Additionally, we found from the NS3h substrate curves for ATP, CTP, GTP and UTP that NS3h displays the same NTP specificity as its full length counterpart (Figure S1 and Table S1).

#### The effect of single stranded RNA on the NS3h ATPase activity

**Figure 3:**
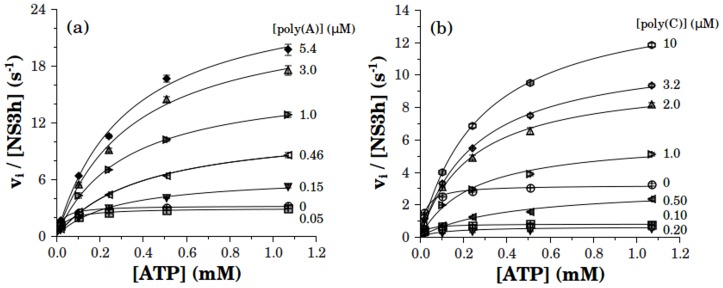
Substrate curves of NS3h in the presence of RNA. Initial rates of ATP hydrolysis were obtained at different concentrations of poly(A) (a) or poly(C) (b). NS3h and RNA were pre-incubated for about 30 to 40 minutes in the reaction media prior to the addition of ATP. Final concentrations of RNA bases are indicated in the plots and final [NS3h] was 10 nM in all experimentsis. Continuous lines are the best fitting hyperbolas for each RNA concentration, with *k_cat_* and *K_M_* values shown in Figure 4.

**Figure 4:**
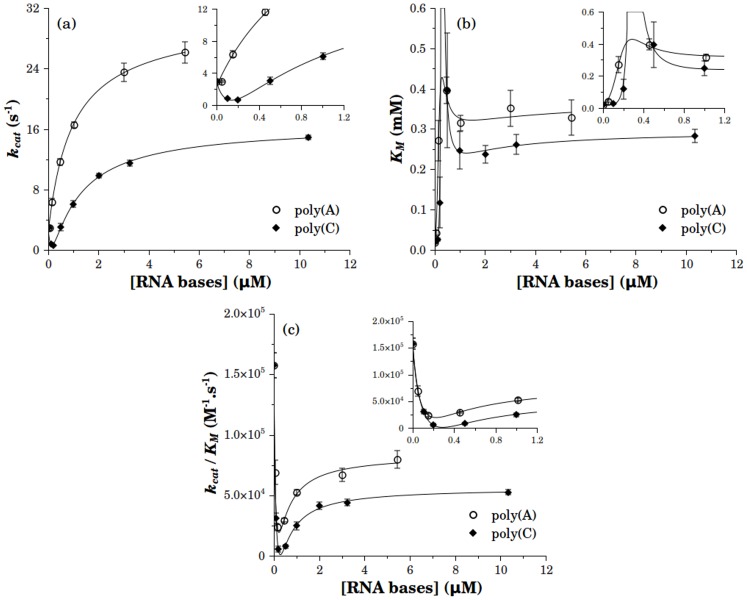
Effect of RNA on the kinetic parameters of the ATPase activity of NS3h. *k_cat_* (a), *K_M_* (b) and *k_cat_/K_M_* (c) values were obtained from substrate curves in Figure 3. In the absence of RNA the values of *k_cat_, K_M_* and *k_cat_/K_M_* were 2.91 (0.03) s-1, 0.018 (0.001) mM and 16 (1) 10^4^ M^-1^s^-1^, respectively. The limit saturating values of *k_cat_, K_M_* and *k_cat_/K_M_* were respectively 28 (1) s^-1^, 0.33 (0.03) mM and 8.4 (0.4) 10^4^M^-1^s^-1^ for poly(A) and 16.6 (0.3) s^-1^, 0.30 (0.02) mM and 5.6 (0.2) 10^4^M^-1^s^-1^ or poly(C). Continuous lines are the best fitting curves to a rational equation and are only intended to guide the eye. Error bars are the standard error of the parameters fitted to the experimental results by nonlinear regression analysis.

To study the effect of RNA on the ATPase activity of NS3 we used polyribonucleotides poly(A) and poly(C). NS3h and RNA were incubated for about 30 to 40 minutes in the reaction buffer, at twice their concentration in the final reaction media. Reactions were started by the addition of an equal volume of ATP solution. We obtained the substrate curves for ATP in the presence of different concentrations of RNA (Figure 3). These curves were well described by equilateral hyperbolas characterized by parameters *k_cat_* and *K_M_* whose values are shown in Figure 4.

The values of *k_cat_* varied monotonically with poly(A) concentration, starting from the basal value to a maximum of 28 s-1 along a hyperbola with a *K_0.5_* of 1.0 M. Instead, the plot of *k_cat_* against poly(C) concentration initially decreased to a minimum of nearly no catalytic activity before a continuous rise to a maximum value of 16.6 s^-1^ (Figure 4a).

*K_M_* values as a function of polynucleotide concentration follow the same trend with poly(A) and poly(C) (Figure 4b). In both cases *K_M_* shows a maximum before reaching a stationary value of 0.33 mM and 0.30 mM for poly(A) and poly(C), respectively. This change in *K_M_* corresponds to a 20-fold increase from its value in the absence of RNA.

The ratio *k_cat_*/*K_M_* (Figure 4c) initially decreases with the concentration of polynucleotide, goes through a minimum and then increases to a stationary value of 8.4×104M^-1^s^-1^ for poly(A) and of 5.6×10^4^M^-1^s^-1^ for poly(C). In both cases, the stationary value was lower than the value obtained in the absence of polynucleotide.

**Figure 5:**
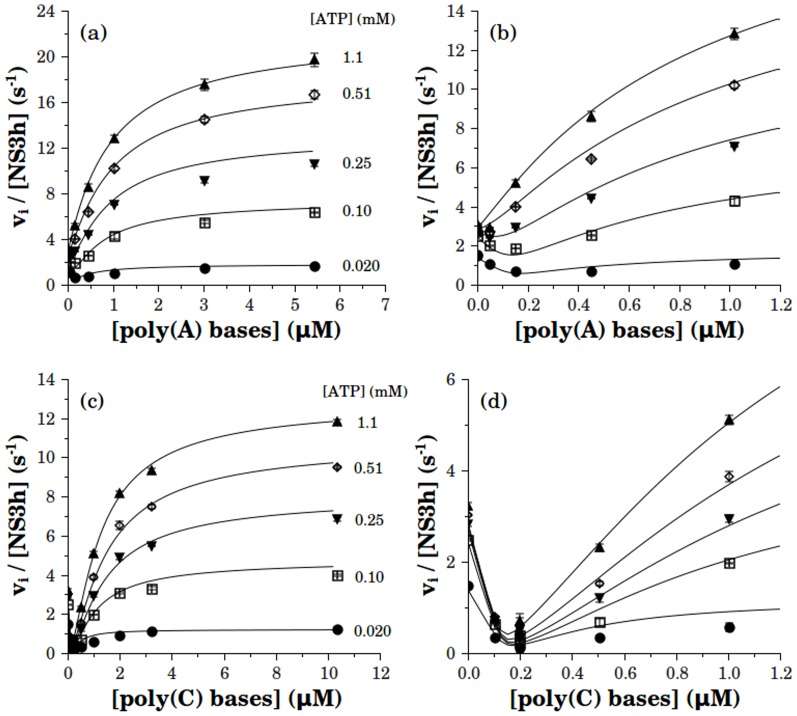
Effect of RNA on the ATPase activity of NS3h. Data showed in Figure 6 are plotted as functions of [poly(A)] (a, b) or [poly(C)] (c, d) at the indicated concentrations of ATP. Continuous lines proceed from simulations of the best fitting solution of the model presented below.

At a given ATP concentration, the addition of RNA may produce either an increase or a decrease in ATPase activity. This can be readily appreciated when the ATPase activity is plotted as a function of polynucleotide concentration (Figure 5). For both, poly(A) and poly(C), the inhibition and activation effects were observed at low and at high concentrations of RNA, respectively. It is worth noting that this same behavior was also observed when the experiments were performed with the full length construct NS3f (Figure S2).

#### ATPase activity of NS3h presents a minimum at 10-20 bases of RNA per NS3 molecule

**Figure 6:**
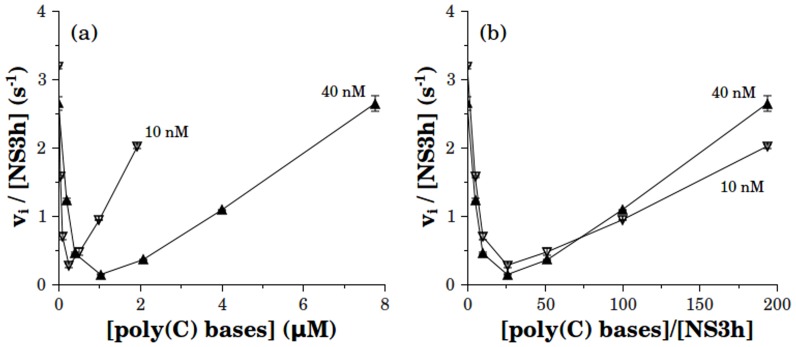
Effect of RNA on the ATPase activity at two different concentrations of NS3h. Initial rates of phosphate release (*vi*) are plotted as a function of [poly(C)] (a) or [poly(C)]/[NS3h] (b). NS3h concentration was 10 nM (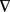) or 40 nM (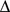). ATP concentration was 0.10 mM and reactions were carried out in the same reaction media as were indicated above (see Materials and Methods).

In order to increase the experimental basis that describes the effect of RNA on the ATPase activity we performed experiments at different concentrations of NS3h. Firstly, the effect of poly(C) concentration on NS3h ATPase activity was measured at two different concentrations of the enzyme (Figure 6a). It can be seen that the ATPase activity curve at the higher NS3h concentration (40 nM) was right-shifted relative to the curve at the lower enzyme concentration (10 nM). When the same data was plotted as a function of the ratio [poly(C) bases]/[NS3h] (Figure 6b) it was found that both curves were superimposed and presented a minimum at a ratio of 20 bases per NS3h molecule. The same behavior was also observed when the experiments were performed with the full length construct NS3f (Figure S3).

**Figure 7:**
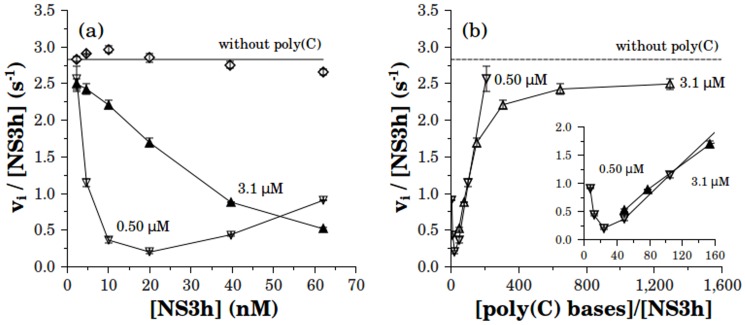
Effect of RNA on the ATPase activity at different concentrations of NS3h. Initial rates of phosphate release (*vi*) were obtained in the absence (♦) and in the presence of poly(C): 0.50 M (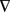) or 3.1 M (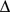). ATP concentration was 0.10 mM and reactions were carried out in the same reaction media as were indicated above (see Materials and Methods). Data showed in (a) as a function of [NS3h] are plotted in (b) as a function of the ratio [poly(C)]/[NS3h].

To validate the experimental consistency of these results the ATPase activity was measured as a function of NS3h concentration at fixed concentrations of poly(C) (Figure 7). The rationale behind this test is that if the so far observed dependence of ATPase activity on the ratio bases/NS3h were no longer found when NS3h concentration changes while RNA is kept constant, then there should be considered the possibility of such dependence being a product of an unspecific effect as could be, for example, any charge effect derived from the addition of polyelectrolyte to the reaction media.

In Figure 7a it can be seen that in the presence of poly(C) ATPase activity initially decreases as a function of NS3h. As expected, the curve obtained at the higher poly(C) concentration (3.1 M) was right-shifted relative to the curve at the lower concentration (0.5 M). It is worth noting that in the limit of high NS3h concentrations, where the amount of RNA becomes negligible relative to that of NS3h, both curves must converge to the value displayed in the absence of RNA, which remained constant in the range of NS3h concentrations tested. When the data was replotted as a function of the ratio [poly(C) bases]/[NS3h] (Figure 7b) it was observed that both curves overlapped and the dependence of ATPase activity on this ratio was the same as that observed in Figures 5 and 6. This fact indicates that the inhibition-activation behavior is dependent on the ratio of bases per NS3 molecule rather than on the individual concentrations of NS3 or RNA.

#### Kinetic model for the effect of RNA on the ATPase activity of NS3

In this section we formulate a minimal kinetic model to predict and explain the results of the experiments on the steady-state ATPase activity of NS3h and its modulation by RNA. The model is built with four experimentally supported assumptions and one hypothesis:

1. NS3 has one binding site for ATP and other for RNA;
2. Each RNA molecule may bind multiple NS3 molecules and each NS3 occludes a fixed number of bases;
3. Products ADP and orthophosphate are released in one step from two different species: an NS3-ATP binary complex and an NS3-RNA-ATP ternary complex;
4. ATP and RNA bind randomly to NS3;

*Hypothesis*. Catalysis of ATP hydrolysis by NS3 bound to RNA is impeded by the presence of other NS3 molecules in the immediate adjacency of the former.

The presence of one binding site for nucleotide and other for RNA in DENV NS3 (assumption 1) is supported by results coming from different studies. One site for each was observed in crystallographic structures [12], [25]. Likewise, the substrates curves of the NTPase activity presented here as well as that reported in [9], [12], [26] are compatible with the presence of only one kinetically distinguishable catalytic site. In addition, functional evidence that NS3 interacts with ssRNA through a single binding site comes from equilibrium binding experiments where we found that binding of short ssRNA (10 to 15 nt long) to NS3 follows a 1:1 stoichiometry with a unique intrinsic association constant (see supplementary Figure S4). Given that the polynucleotides used here are on average much longer than the number of bases that could be covered by a single NS3 molecule (see Material and Methods and [18]), the model considers that multiple NS3 molecules may bind along a single polynucleotide chain, as was stated in assumption 2.

Product release from an enzyme-substrate-modifier ternary complex, in addition to that from an enzyme-substrate binary complex, (assumption 3) is a minimal requirement for a model to give a saturating curve as a function of modifier concentration (see Figure 5). Note that if no such ternary complex capable of product release were present, all those curves would go asymptotically to zero as modifier concentration is increased. This consideration implies that the formation of a ternary complex NS3-RNA-ATP is a necessary assumption which is in agreement with crystallographic structures showing NS3 bound to both RNA and ATP analogs [18], and along with the experimental evidence that RNA binds to NS3 in the absence of ATP [20](manuscript in preparation) and that ATP binds to NS3 in the absence of RNA allow us to assume a random mechanism of ligand addition to the NS3 (assumption 4).

Random binding (assumption 4) and product release from both enzyme-substrate and enzyme-substrate-modifier complexes (assumption 3) are characteristic features of general steady-state kinetic models involving one substrate and one modifier [27]–[30] which we refer to as classical modifier models. These models are general in that they consider that the modifier may bind to all of the enzyme-substrate intermediates with no necessarily equal affinities and modify the reaction rates along the catalytic cycle. Given that a kinetic model of this sort provided a poor fit to the experimental results shown above (see Discussion) it was necessary to introduce one additional assumption. This was accomplished considering that the ATPase activity of DENV NS3h is impeded by the presence of other NS3 molecules in the immediate adjacency.

The rationale behind this hypothesis is the following. As can be observed in Figures 5–7, ATPase activity first decreases from its basal value as polynucleotide was added till its concentration reaches the value corresponding to a ratio of about 20 bases per NS3 molecule. After that, a sustained increase follows up to saturation. Keeping in mind that such ratio value is close to the 8–12 bases occluded in the NS3 observed in crystallographic studies [18], it appears that the mentioned increase in ATPase activity takes place only after the concentration of bases exceeds the value needed to fill up all of the RNA binding sites present at a given NS3 concentration. In the same way, the results shown in Figures 5–7 suggest that, when NS3 is in excess over the number of bases available for binding and RNA chains are highly covered by NS3 molecules, association to RNA leads to inhibition of its ATPase activity. Given that binding under such conditions is associated to a high binding density of NS3 molecules on the RNA lattices (see below) we formulated the hypothesis stated above.

In order to implement the hypothesis into a kinetic model it will be considered that NS3 may bind to three kinds of binding sites on the RNA: (i) surrounded by free bases on both sides or *isolated*; (ii) with a free base on one side and an occluded base on the other or *singly contiguous*; or (iii) surrounded by occluded bases on both sides or *doubly contiguous*. Therefore there will be four populations of NS3 molecules bound to RNA: one associated to isolated sites, one associated to doubly contiguous sites and two other populations associated to singly contiguous sites, flanked on the 5′ or the 3′ side by another NS3 molecule. As will be explained later, the distribution of NS3 molecules bound to RNA between these populations will be computed employing the formalism developed by McGhee *et. al.* [22] for the analysis of the binding of large ligands to one-dimensional lattices of infinite length. Then, each of these populations will be assigned to one of two states of NS3 bound to RNA: one able to catalyze the hydrolysis of ATP (*uncrowded state*) and other without catalytic activity (*crowded state*).

**Figure 8:**
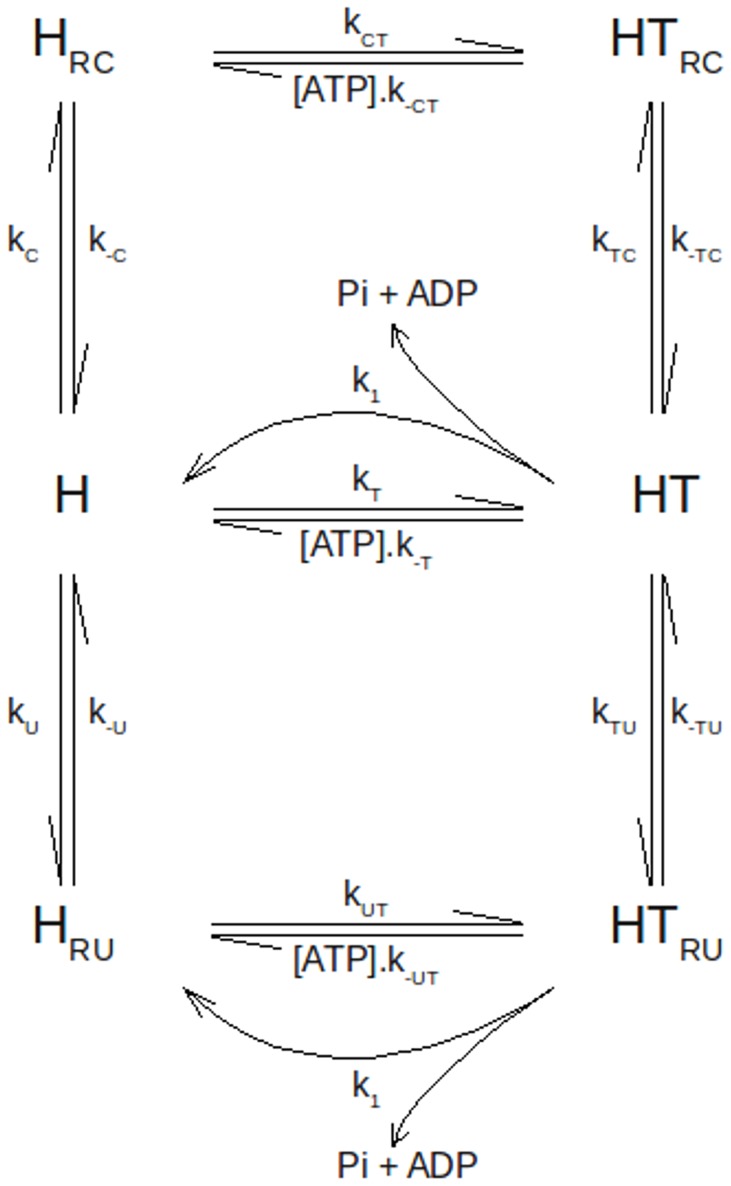
Kinetic model for the interaction between NS3 and RNA. H is NS3, T is ATP, Pi is orthophosphate, HRC is NS3 bound to RNA in a crowded state and HRU is NS3 bound in an uncrowded state. ADP release is supposed to be simultaneous to Pi release.

A scheme of a kinetic model constructed upon the assumptions described above is shown in Figure 8. The model involves six NS3 ligation states: free (H), bound to ATP (HT), bound to RNA in a crowded or an uncrowded state (H_RC_ or H_RU_, respectively) and bound to both ATP and RNA in a crowded or an uncrowded state (HT_RC_ and HT_RU_).

According to this reaction scheme the rate of Pi release is given by:
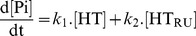
(4)where *k_1_* and *k_2_* are the rate constants for product release from HT and HTRUcomplexes, respectively. In terms of this kinetic model, these rate constants are the turnover constants of free NS3 (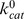) and of NS3 bound to RNA in an uncrowded state (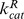), respectively.

*k_U_* , *k_C_* , *k_TU_* and *k_TC_* are apparent first order rate constants defined as:
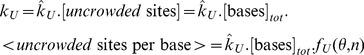
(5)

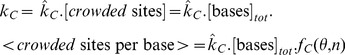
(6)

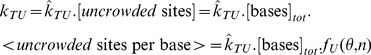
(7)

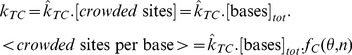
(8)where 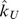, 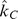, 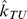 and 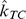 are the second order rate constants for the association of NS3 to RNA; [*crowded* sites] and [*uncrowded* sites] are the concentration of free crowded and uncrowded sites, respectively; [bases]*_tot_* is the total concentration of polynucleotide bases. The number of crowded and uncrowded free sites per base are provided by *f_C_(* 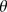, *n)* and *f_U_*(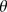, *n)*, respectively, which in turn are functions of *n* -the number of contiguous bases on the RNA occluded by one NS3 molecule- and 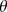 -the number of bound NS3 molecules per RNA base (see Supporting Information Text S1).

Parameters of the kinetic model of Figure 8 were fitted to the experimental data by non-linear regression analysis (see Material and Methods). As explained in the Supporting Information Text S1, the fitting was done upon three different assignments to the kinetic model:

1. only NS3 molecules bound to isolated sites are in an uncrowded state,
2. only those bound to doubly contiguous sites are in a crowded state;
3. NS3 molecules bound to isolated sites in addition to NS3 molecules bound to one half of singly contiguous sites are in an uncrowded state.

Each of these possibilities was assessed including a cooperativity parameter (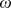) that affects the interaction between NS3 and RNA (see Supporting Information Text S1). The value of this parameter was either fixed as 1 or left free during the fitting process.

The best description of the results was obtained applying the assignment (3) without cooperativity (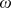  =  1) to the kinetic model described above. The continuous lines in Figures 5–7 are simulations of the experimental results obtained using the best fitting parameter values shown in Table 2.

**Table 2:** Best-fitting values of the model in Figure 8 (SE).

Parameter	without RNA	poly(A)	poly(C)	units
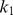	2.91 (±0.03)	–	–	s^-1^
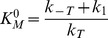	0.018 (±0.001)	–	–	mM
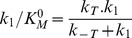	16 (±1)	–	–	10^4^M^-1^s^-1^
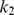	–	26.6 (±0.3)	15.5 (±0.1)	s^-1^
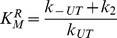	–	0.22 (±0.02)	0.203 (±0.005)	mM
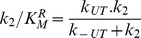	–	12 (±1)	8 (±1)	10^4^M^-1^s^-1^
n	–	–*	10.9 (±0.3)	bases per NS3
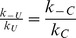	–	23 (±18)	14 (±24)	nM
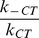	–	0.15 (±0.32)	0.29 (±1.39)	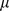 M

* The value of occluded site size (*n*) was obtained from fitting to the results in the presence of poly(C) and was used for the fitting to the results in the presence of poly(A) (see Material and Methods).

## Discussion

### NTPase specificity

Previous studies on dengue virus NS3 protein had already shown that it catalyzes the hydrolysis of all four canonical NTPs (ATP, CTP, GTP and UTP) [8], [9], [31] and that this NTPase activity is stimulated by ssRNA. The ability to discriminate between different NTPs (specificity) was also partially assessed in some of those studies [9], [10], [21], [31]. Here, the specificity of NS3 for those nucleotides was evaluated according to the ratio *k_cat_/K_M_* (Figures 1 and S1 and Tables 1 and S1). It was found that both the full length and the isolated helicase domain of NS3 show poor specificity to discriminate between these nucleotides. The promiscuity of NS3 toward NTP substrates and its poor specificity is in accordance with the observation from crystallographic studies that the ribose and base moieties of ATP analogs bound to DENV NS3 might be exposed to the solvent, whereas the triphosphate appears to be buried between the RecA-like motor domains [32] and could be associated to the lack of a conserved Q motif in DExH NS3 helicases, which appears to mediate contacts with the adenine base of ATP in the more specific DEAD-box helicases [33], [34].

An additional observation is that the values of *k_cat_/K_M_* -in the order of 10^4^–10^5^ M^-1^s^-1^ - which represent the apparent second order rate constant for the formation of the enzyme-substrate complex, are far below that expected for a diffusion-controlled bimolecular reaction (10^8^–10^9^ M^-1^s^-1^). In terms of the molecular mechanism of NTP hydrolysis catalyzed by NS3, this fact indicates that if there is a rate-controlling step of the catalytic cycle it is located after any diffusional process along the reaction pathway [35].

### NTPs are hydrolyzed in a same catalytic site

A further question regarding the NTPase activity of NS3 is whether all substrates are hydrolyzed in a same or in different catalytic sites. Several results presented in previous works were compatible with the idea that only one active site was involved in NTPase activity as well as in 5′-RTPase activity [10], [21], [32]. We examined this issue by performing competition experiments in which two NTP substrates were present at a time in the reaction media. The competition plots obtained in this way presented no maxima at non extreme *x* values (Figure 2) indicating that the presence of one of the nucleotides inhibits the reaction of the other [24]. These results are compatible with different kinetic models involving either:

1. a unique catalytic site for all substrates;
2. multiple identical and independent catalytic sites for all substrates;
3. one catalytic site for each substrate with cross inhibition either when (a) each substrate has a *K_M_* value equal to that of the *K_0.5_* for the inhibitory effect on the other site or (b) when the substrate concentrations were casually chosen in such a way that simplifications of terms on the rate equation yield the same expression as those obtained in models (1) and (2), even though the reactions are not truly competitive [24].

Now, in order to assign one of these possibilities to the steady-state NTPase activity of NS3, we formulate the following considerations. The cross inhibition alternative (3a and 3b) is unlikely to be the cause of the observed results because this would require that the fortuitous relations mentioned in 3a and 3b happen to be true for each pair of substrates tested. Regarding the other two possibilities (1) and (2) it must be noted that our results do not provide any information to distinguish between them. This is because velocity measurements under steady state conditions can not differentiate between kinetic models involving one or multiple identical and independent catalytic sites. The only information available about this subject comes from crystallographic studies, where it was shown that ADP and ATP analogs appear to bind to a unique binding site in all resolved structures [18]. Taken together with our results, all the information available supports the notion that a single catalytic site on the NS3 of dengue virus accounts for its NTPase activity.

### The effect of RNA on the ATPase activity of NS3

A general conclusion that can be drawn from the literature is that the NTPase activity of helicases is stimulated upon binding to nucleic acids. However, in some cases, inhibition effects at low ATP [36] as well as non monotonous dependence of the ATPase activity on nucleic acid concentration [37] were observed. In this context, the results presented here show that the value of the catalytic constant (*k_cat_*) at saturating RNA is about ten times greater than in the absence of RNA and that the effect of RNA on the ATPase activity was non monotonous at low ATP concentrations (Figures 3–6).

In the search for a minimal kinetic model that could account for the experimental results, we analyzed the classical modifier model, since it is able to give rise to both inhibition by the modifier at low substrate concentrations and a non monotonous dependence of enzymatic activity on modifier concentration [27], [28], [30]. Nevertheless, as was stated above, fitting of the classical modifier model to the experimental results was unsuccessful. Particularly, the non monotonous dependence of *k_cat_* on poly(C) concentration can not be predicted by such model. Instead, it predicts a monotonous increase of *k_cat_* up to saturation. Secondly and more generally, global fitting of the classical modifier model to all the data resulted in serious bias. All best fitting set of parameters predicted a monotonous dependence of ATPase activity on RNA concentration; that is, albeit they did predicted inhibitory effects –principally at low ATP concentrations- inhibition was never followed by an increase on ATPase activity. These observations suggested us that NS3 is more strongly inhibited –at the so far mentioned values of the ratio bases/NS3- than what the classical modifier model could predict.

An important theoretical framework to analyze the effect of nucleic acids on the steady-state ATPase activity of helicases was developed by Young *et.al.* [38] and was recently applied by Rad *et.al.* [39]. In this formalism the dependence of ATPase activity -at a fixed ATP concentration- on the length of the nucleic acid is associated to the translocation property of helicases. There are several reasons why we could not apply this formalism to the analysis of our results. For instance, the kinetically relevant inhibition of ATPase activity at low bases per NS3 ratios is observed in conditions in which the Young's formalism can not be applied. Namely, the requirement that the total concentration of lattice binding sites is much greater than the enzyme concentration is not fulfilled.

Without invoking any activation mechanism and backed up by equilibrium binding studies Wong *et. al.* have successfully explained the complex dependence of ATPase activity of *E. coli* Rep helicase on DNA concentration in terms of the distribution of the enzyme between different Rep-DNA ligation sates [37]. There the authors demonstrated that, if a helicase presents oligomeric states that can give rise to different ligations states upon binding to nucleic acids, complex dependence of steady-state ATPase activity on nucleic acid concentration is to be expected as a result of differences on the ATPase activity displayed by each ligation state. For DENV NS3, however, we have evidence that such explanation cannot be applied. Results coming from light scattering and equilibrium binding experiments revealed that NS3h is a monomeric protein that binds to short ssRNA with 1:1 stoichiometry and a unique intrinsic association constant (see supplementary Figure S4).

The kinetic model shown in Figure 8, built on the basis of the considerations described above, was able to describe the effect of RNA concentration on the ATPase activity of DENV NS3. This model predicts that the value of the RNA-NS3 equilibrium dissociation constant is about 10 nM, which is close to the value obtained from equilibrium binding experiments (manuscript in preparation). Additionally, notwithstanding that RNA produced an increase on the *K_M_* value –a decrease on the apparent affinity- for the ATPase activity substrate curves (Figure 4) the model predicts that the affinity for ATP increases with the binding of RNA to NS3h (Table 2 and 1). This effect of RNA on the affinity of NS3 for ATP is opposed to what was found for the hepatitis C virus NS3 [36], [40], [41] and requires verification from equilibrium binding experiments. Finally, it is worth noting that the value of the ‘occluded site size’, *n*, proceeding from the fittings of the model to the experimental results (11 bases per NS3) was very close to the value inferred from crystallographic studies [18].

In order to explain our results and on the basis of the considerations described above(see Kinetic model for the effect of RNA on the ATPase activity of NS3), we put forward the assumption that inhibition at low bases/NS3 ratios was the result of crowding of NS3 molecules along the RNA lattices. In the kinetic model presented above inhibition by crowding implies that NS3 bound to a given RNA lattice affects the activity of other NS3 molecules bound to the same lattice. We can envisage at least three molecular mechanisms through which NS3 molecules may affect the ATPase activity of other NS3 molecules located on its neighborhood: (1) through hindering of the ATPase catalytic site by close lateral proximity of NS3 molecules, (2) through allosteric changes on the ATPase catalytic site induced by the interaction between adjacent NS3 molecules or (3) through steric blockage of conformational transitions involved in the ATPase catalytic cycle.

Each one of these possibilities can be identified with some of the assignments for the crowded and uncrowded states formulated above: the first, with the kinetic models in which NS3 is inactive when bound to doubly contiguous and one half of single contiguous sites on the RNA lattice (assignment (3)); the other two, with any of the three assignments albeit, in the second case, there must be added a cooperativity parameter affecting the binding of NS3 to RNA.

Regarding the molecular mechanism (1), available crystallographic structures of NS3 bound to RNA shows that the nucleotide binding site of dengue virus NS3 opens almost perpendicularly to the direction of the RNA chain [18]. This observation is difficult to conciliate with the occlusion of the ATPase catalytic site generated by the lateral proximity of adjacent NS3 molecules.

The second possible molecular mechanism (2), involves allosteric effects between the ATPase catalytic site and the putative NS3-NS3 interaction promoted by lateral proximity. Now, given that the ATPase catalytic site is allosterically linked to the RNA binding site, the allosterism with such NS3-NS3 interaction should manifest too on this later RNA binding site. This would be observed as cooperativity (negative or positive) in the binding of multiple NS3 molecules on the RNA, such as was observed in many other helicases (surveys can be found in [42] and [43]). We introduced and fitted a cooperativity parameter into the tested kinetic models under the three possible assignments. This parameter did not improve significantly the fitting to the experimental results and its value was poorly defined.

A particular case representative of the last molecular mechanism is one in which translocation of a given NS3 molecule along the RNA is blocked by adjacent NS3 molecules and such blockage interrupts the progress of the ATPase catalytic cycle. This form of inhibition would then be a manifestation of tight coupling between the ATPase catalytic cycle and the translocation of NS3 along the RNA lattice. Tight coupling between ATPase activity and translocation means that any of these processes may work only as the other process works and is a characteristic property of the stepping mechanisms of helicases described in the literature [44], [45]. In this regard, there are experimental evidence both in favor and opposed to this idea. Most if not all of the results in favor of this kind of mechanisms come from the observation of the number of ATP molecules hydrolyzed in each translocation –or unwinding- step (see for example [46], the first direct measurement of the coupling efficiency of a helicase; see also [47], [48]). In the context of the kinetic model presented above, assignment (3) is the implementation of the possibility that inhibition of a given NS3 molecule is only produced by adjacent proteins on one of its sides and thus may be regarded as the representation of a mechanism of inhibition by translocation blockage. Naturally, the results presented in this work does not provide an answer to whether DENV NS3 works through a tightly coupled mechanism, but given the relevance of this issue in understanding the energy transduction process performed by helicases, it is worthwhile to explore further the possibility that measurements of the effect of long single stranded nucleic acids on the steady-state ATPase activity provide valuable information on such question.

## Materials and Methods

### Reagents

MOPS, NaCl, KCl, KOH, HCl, KH_2_PO_4_ and MgCl_2_ were from Mallinckrodt Baker (ACS reagents). Ascorbic acid was from Anedra. EDTA tetraacid, nucleoside triphosphates (ATP, CTP, GTP and UTP, sodium salts), sodium meta-arsenite and polyadenylic acid (poly(A), potassium salt, Catalog number P9403) were from Sigma Aldrich. Sodium citrate and ammonium heptamolybdate were from Merck. Polycytidylic acid (poly(C)) was from Amersham (potassium salt, product number 27-4220, lote number 4084220021).

According to the average sedimentation coefficient reported by the manufacturer (S_20_ w 11, in 15 mM NaCl, 1.5 mM sodium citrate, pH 7.0), the poly(C) preparation has an average chain length of about 1200 nucleotides.

Stock solutions of NTPs and polynucleotides were prepared in RNase-free ultrapure water and buffer and their base concentrations were determined spectrophotometrically [49].

### Protein constructs and purification

Constructs encoding NS3 proteins were derived from the cDNA of an infected clone of DENV serotype 2 (Gene Bank accession number U87411) [50]. The previously obtained plasmids pET-NS3-hel and pET-CF47-NS3-H51A were used for high level expression of N-terminal hexa-histidine tagged recombinant proteins in *E. coli* [11]. The pET-NS3-hel expression construct encodes for the NS3 helicase domain (residues 171–618) in vector pET-28a (Novagen, Madison, USA). The pET-CF47-NS3-H51A plasmid was assembled as the 47-amino-acid hydrophilic core sequence of NS2B (amino acids 49–95) linked via a nine-amino-acid linker (GGGGSGGGGG) to the full-length sequence of NS3 (amino acids 1–618). In order to avoid auto-proteolytic degradation of the resulting protein, histidine at position 51 of the catalytic triad of the protease domain was replaced for alanine. Protein expression and purification procedures were identical as described in a previous report [11]. Homogeneity of protein preparations was assessed by SDS-PAGE and Coomassie Brillant Blue staining. The concentration of enzyme in stock aliquots was calculated by the Edelhoch method from its empirical extinction coefficient (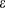
_280_  =  73134 M^-1^ cm^-1^ for NS3h and 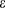
_280_  =  108250 M^-1^ cm^-1^ for NS3f) and its absorbance at 280 nm.

In addition, protein mass content computed from molecular weights and molar concentrations obtained in this way, gave the same value as that determined by Bradford's assay [51].

### Determination of inorganic phosphate

Release of inorganic phosphate was monitored spectrophotometrically using the method of Baginski and collaborators [52] with modifications. This procedure is based on the formation of blue-colored complexes between phosphate and molybdate under reductive conditions. To sample volumes ranging between 250 to 300 µl, equal volumes of reagent A (composed by 0.5% w/v ammonium heptamolybdate and 3% w/v ascorbic acid in 0.5 N HCl at approximately 4°C) were added and the resulting mixtures were incubated in an ice-water bath. After 10 to 20 minutes, 450 or 600 l of reagent B (composed by 2% w/v sodium citrate, 2% w/v sodium arsenite and 2% v/v acetic acid) was added and mixtures were incubated during 10 to 20 minutes at 37 °C. Finally, after approximately 30 minutes at room temperature their absorbances at 850 nm were recorded. Phosphate concentration in reaction mixtures were evaluated by interpolation in calibration curves (one for each experiment) obtained from phosphate standard solutions in the same media. It was checked that the absorbance dependence on phosphate concentration was not significantly changed upon the addition of our preparations of proteins, NTPs or RNAs.

### General experimental conditions

All reactions were carried out at 25°C in media containing 25 mM MOPS (pH 6.5), 0.5 mM EDTA and enough MgCl_2_ to give a final concentration of free Mg^2+^of 1.5 mM. The equilibrium dissociation constants used to compute the amount of MgCl_2_ were taken from the literature [53] (at pH 6.5 and ionic strength 0.10 M, Kd  =  83 M for ATP, 80 M for CTP, 56 M for GTP and 70 M for UTP).

In addition to these components, 100 mM KCl and glycerol 3% v/v was added in the reactions with the full length protein NS3f which concentration was 50 nM in all experiments. For the reactions with the helicase domain NS3h there was 20 mM KCl, 10 mM NaCl and 0.1 % w/v Tween-20. Otherwise indicated, NS3h concentration was 10 nM.

### NTPase activity measurement

The initial rate of NTP hydrolysis was obtained from the slope of the linear time course of phosphate release. Reaction blanks with denatured enzymes were made to evaluate non-enzymatic NTP hydrolysis which was found to be negligible during the reaction times tested. All reactions were started with the addition of enzyme or NTP to the media and were stopped by the addition of reagent A. Otherwise stated, at least four reaction times were used to evaluate the initial rates of NTP hydrolysis.

When RNA was present in the reaction media, it was incubated with NS3 for 30 to 40 minutes. This time was long enough to achieve a chemical equilibrium condition prior to the start of the reaction with the addition of ATP.

### Data analysis

Libreoffice 3.4.4 spreadsheets were employed throughout our work to compute initial rates of NTP hydrolysis and to fit and simulate substrate curves and competition plots (see Results). The kinetic model for the effect of RNA on ATPase activity was built in Copasi version 4.7 (University of Virginia) which was used to obtain global fittings and simulations [54].

## Supporting Information

Figure S1 **Steady-state NTPase activity of NS3h as a function of substrate concentration.** Initial rates of NTP hydrolysis were obtained for ATP (empty diamond), GTP (filled circle), CTP (filled square) and UTP (filled diamond). The experiments were performed using 10 nM NS3h in a reaction media as indicated in Materials and Methods. Continuous lines are plots of hyperbolic functions whose parameter values (*k_cat_* and *K_M_*) were obtained by non-linear regression analysis and are shown in Table S1.
(TIFF)

Figure S2 **Effect of RNA on the ATPase activity of NS3f.** Initial rates of phosphate release (*vi*) are plotted as a function of [ATP] (a) and as a function of [poly(C)] (b and c). NS3f and RNA were pre-incubated for 40 minutes in the reaction media prior to the addition of ATP. Concentrations of RNA bases and ATP are indicated in the plots and final [NS3f] was 10 nM. Substrate curves in (a) were well described by equilateral hyperbolas, such as was observed for the isolated helicase domain (Figure 3). In (b) and (c) it is observed the characteristic nonmonotonous behavior observed for NS3h in Figure 5, that is, an initial inhibition followed by activation as RNA concentration was increased. Reactions were carried out in the same reaction media as in Figure 3.
(TIFF)

Figure S3
**Effect of RNA on the ATPase activity at two different concentrations of NS3f.** Initial rates of phosphate release (*vi*) are plotted as a function of [poly(C)] (a) or [poly(C)]/[NS3f] (b). NS3f concentration was 10 nM (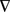) or 40 nM (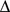). ATP concentration was 0.10 mM and reactions were carried out in the same reaction media as in Figure 3. It can be seen that effect of the ratio [poly(C)]/[NS3f] on the ATPase activity shown here for NS3f is the same as that shown in Figure 6 for NS3h.(TIFF)

Figure S4
**Binding of NS3h to single stranded RNA.** Titration of fluorescein-labeled 10nt long RNA with NS3h was monitored by the fluorescence intensity emitted upon excitation at 495 nm. Relative fluorescence enhancement was computed as (F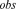-F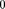)/F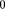, where F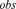 denotes the fluorescence intensity observed at the given NS3h concentration and F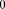 is the fluorescence intensity observed in the absence of protein, and both quantities were measured as the total intensity emitted between 525 and 570 nm minus the intensity recorded in the absence of RNA. Sequence of RNA was 5′-fluo-AGUUGAGUUG-3′. Reaction media and temperature were the same as that employed for the ATPase activity measurements (see Materials and Methods). Continuous lines proceed from the simulation of the best fitting solution of a single-site binding model with 1:1 stoichiometry and a K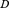 of 0.75 0.08 nM.(TIFF)

Table S1 **Parameters of the steady-state NTPase activity of NS3h in the absence of RNA.**
(PDF)

Text S1 **Deduction of the number of isolated (** ***iso*** **), one-side contiguous (** ***1s*** **) and two-side contiguous (** ***2s*** **) sites per base.**
(PDF)
