# Supplementary figures and images for: Steady-State NTPase Activity of Dengue Virus NS3: Number of Catalytic Sites, Nucleotide Specificity and Activation by ssRNA
Source: PLoS One. 2013 Mar 19;8(3):e58508. doi: 10.1371/journal.pone.0058508 (PMC3602377; doi:10.1371/journal.pone.0058508)

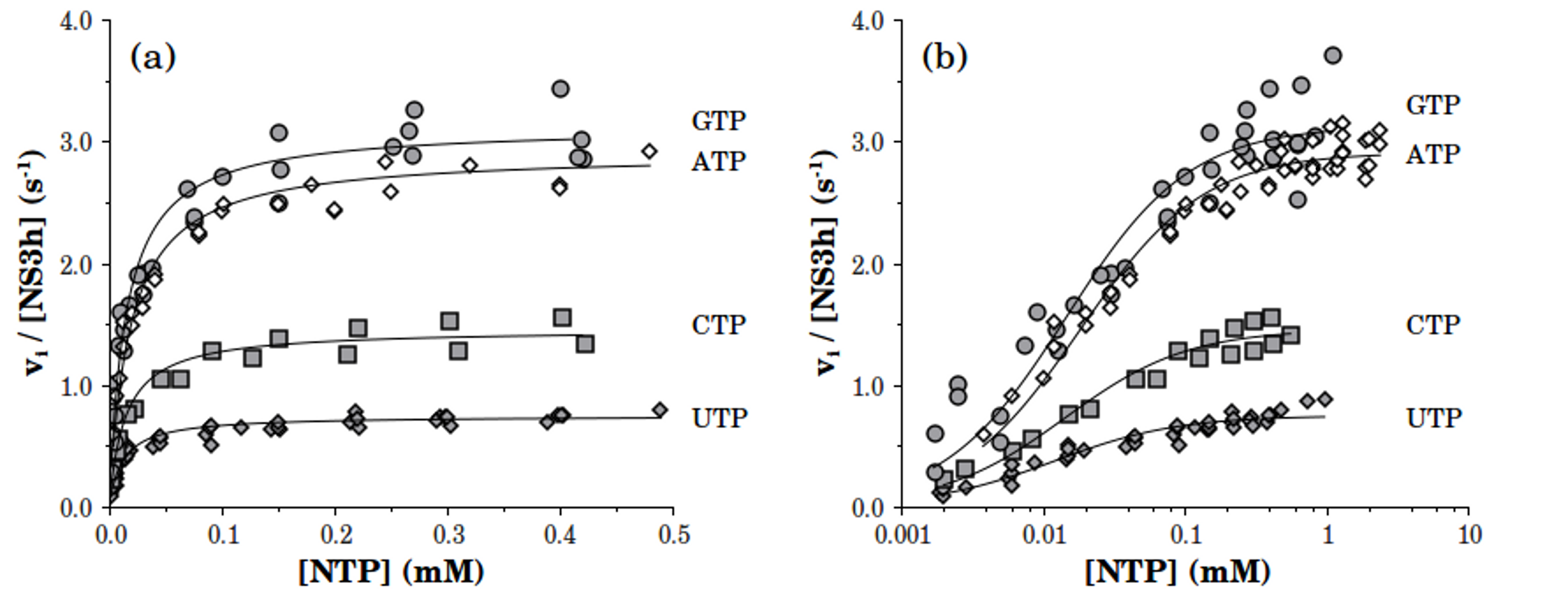

Supplement: Figure S1 — Steady-state NTPase activity of NS3h as a function of substrate concentration. Initial rates of NTP hydrolysis were obtained for ATP (empty diamond), GTP (filled circle), CTP (filled square) and UTP (filled diamond). The experiments were performed using 10 nM NS3h in a reaction media as indicated in Materials and Methods. Continuous lines are plots of hyperbolic functions whose parameter values (kcat and KM) were obtained by non-linear regression analysis and are shown in Table S1. (TIFF) [file pone.0058508.s001.tiff]

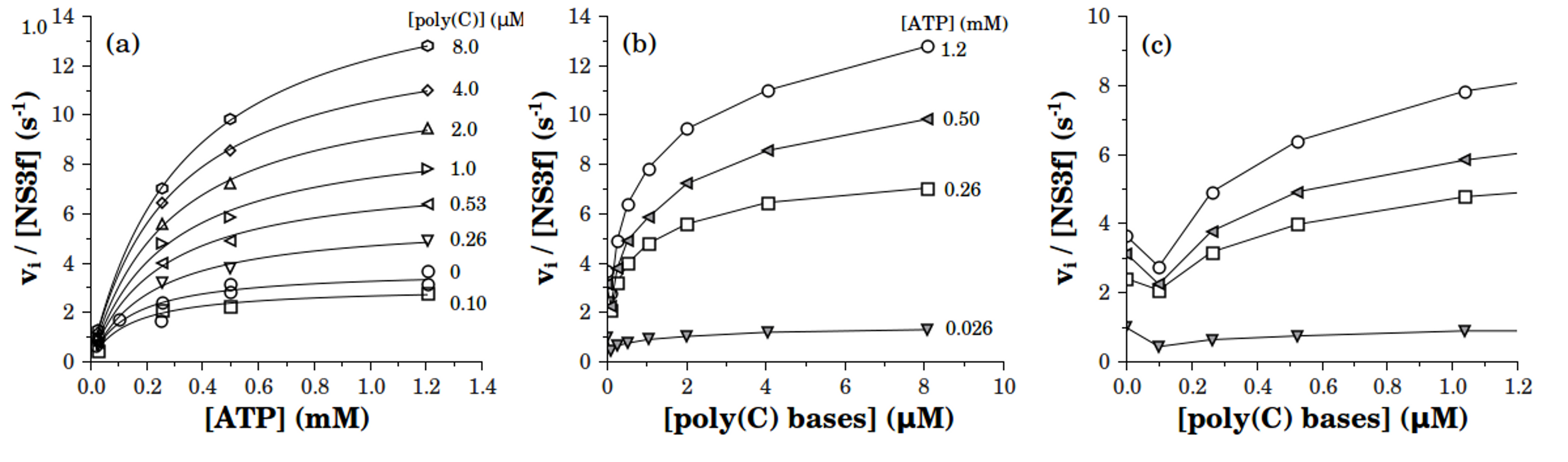

Supplement: Figure S2 — Effect of RNA on the ATPase activity of NS3f. Initial rates of phosphate release (vi) are plotted as a function of [ATP] (a) and as a function of [poly(C)] (b and c). NS3f and RNA were pre-incubated for 40 minutes in the reaction media prior to the addition of ATP. Concentrations of RNA bases and ATP are indicated in the plots and final [NS3f] was 10 nM. Substrate curves in (a) were well described by equilateral hyperbolas, such as was observed for the isolated helicase domain (Figure 3). In (b) and (c) it is observed the characteristic nonmonotonous behavior observed for NS3h in Figure 5, that is, an initial inhibition followed by activation as RNA concentration was increased. Reactions were carried out in the same reaction media as in Figure 3. (TIFF) [file pone.0058508.s002.tiff]

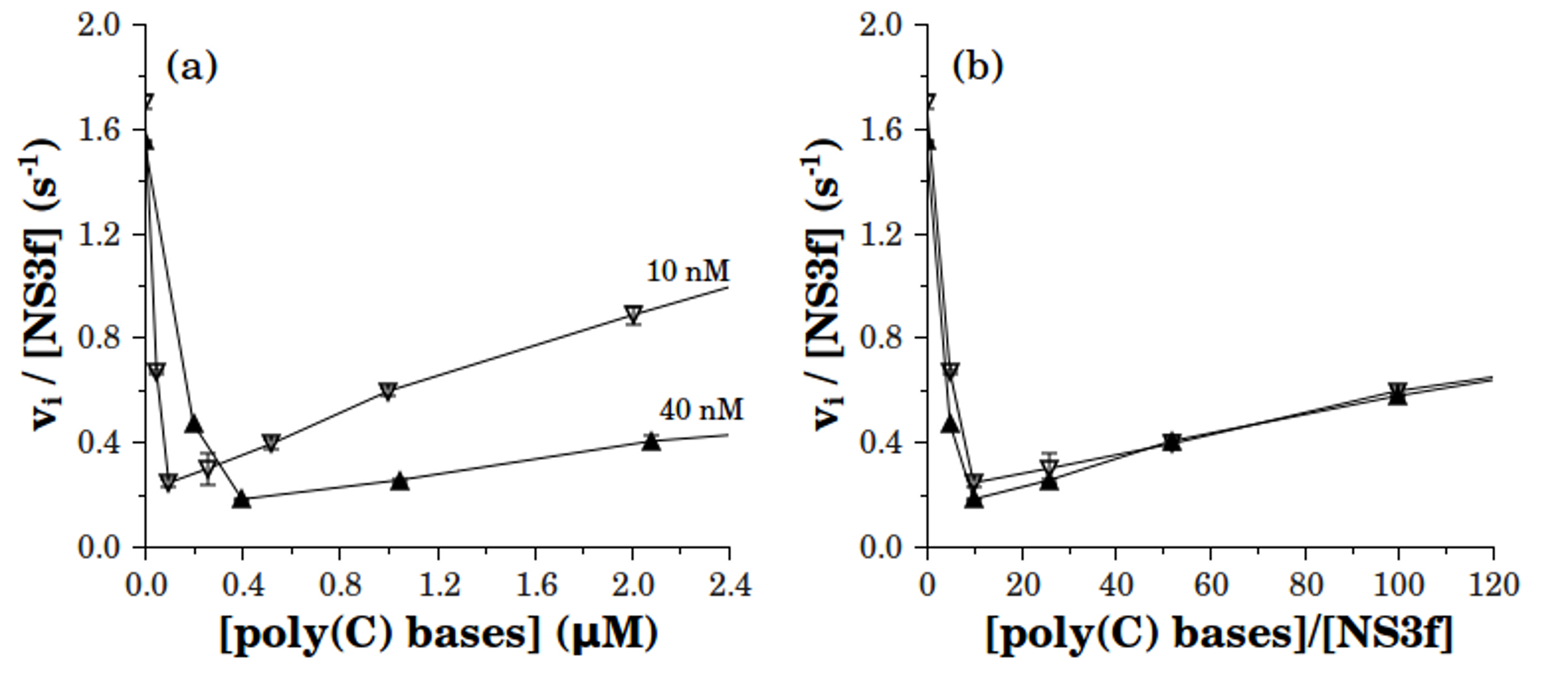

Supplement: Figure S3 — Effect of RNA on the ATPase activity at two different concentrations of NS3f. Initial rates of phosphate release (vi) are plotted as a function of [poly(C)] (a) or [poly(C)]/[NS3f] (b). NS3f concentration was 10 nM () or 40 nM (). ATP concentration was 0.10 mM and reactions were carried out in the same reaction media as in Figure 3. It can be seen that effect of the ratio [poly(C)]/[NS3f] on the ATPase activity shown here for NS3f is the same as that shown in Figure 6 for NS3h. (TIFF) [file pone.0058508.s003.tiff]

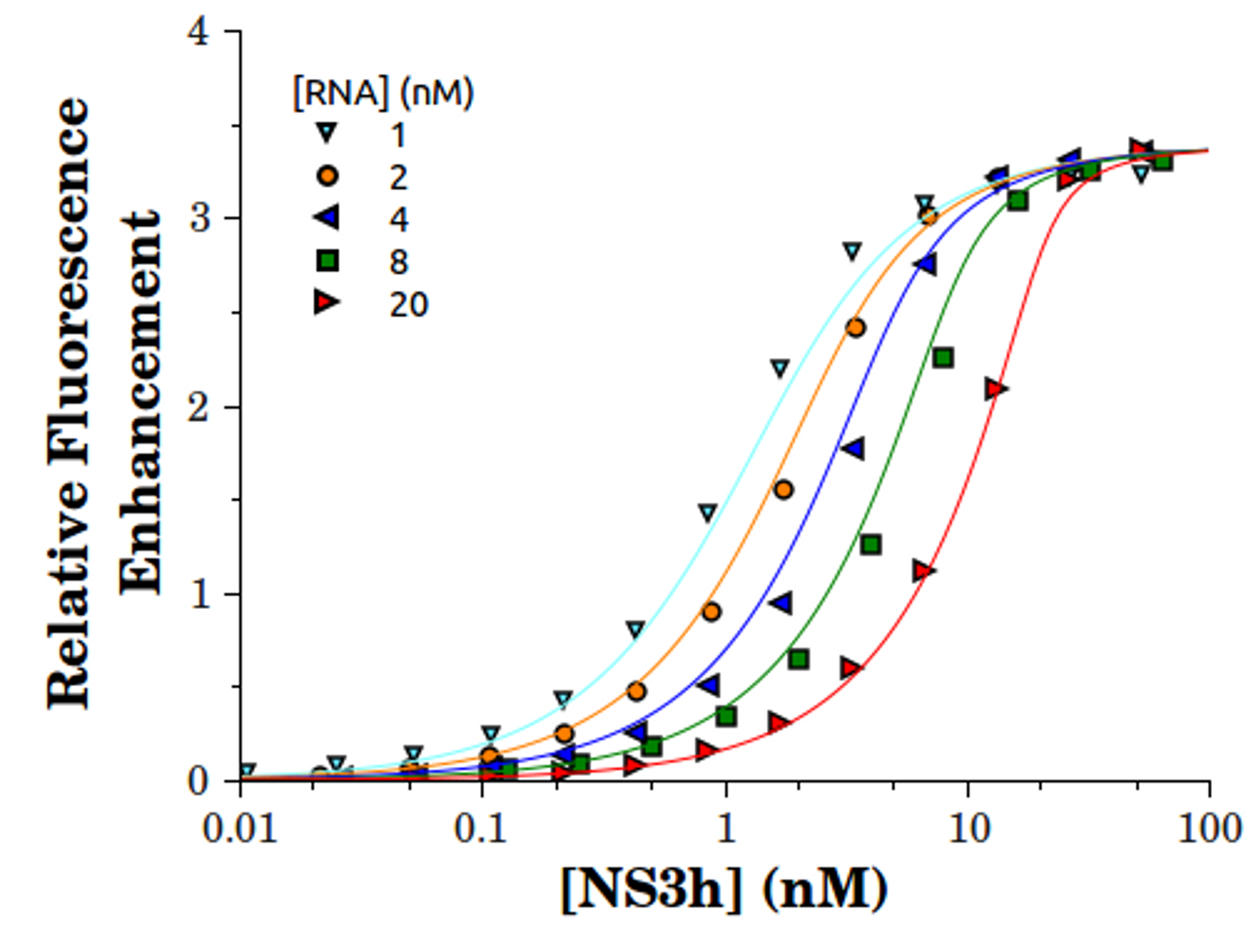

Supplement: Figure S4 — Binding of NS3h to single stranded RNA. Titration of fluorescein-labeled 10nt long RNA with NS3h was monitored by the fluorescence intensity emitted upon excitation at 495 nm. Relative fluorescence enhancement was computed as (F-F)/F, where F denotes the fluorescence intensity observed at the given NS3h concentration and F is the fluorescence intensity observed in the absence of protein, and both quantities were measured as the total intensity emitted between 525 and 570 nm minus the intensity recorded in the absence of RNA. Sequence of RNA was 5′-fluo-AGUUGAGUUG-3′. Reaction media and temperature were the same as that employed for the ATPase activity measurements (see Materials and Methods). Continuous lines proceed from the simulation of the best fitting solution of a single-site binding model with 1:1 stoichiometry and a K of 0.75 0.08 nM. (TIFF) [file pone.0058508.s004.tiff]
