# Supplementary material for: Steady-State NTPase Activity of Dengue Virus NS3: Number of Catalytic Sites, Nucleotide Specificity and Activation by ssRNA
Source: PLoS One. 2013 Mar 19;8(3):e58508. doi: 10.1371/journal.pone.0058508 (PMC3602377; doi:10.1371/journal.pone.0058508)
Supplement: Table S1 — Parameters of the steady-state NTPase activity of NS3h in the absence of RNA. (PDF) [file pone.0058508.s005.pdf]

Table S1. Parameters of the steady-state NTPase activity of NS3h in the absence of RNA.

NTP	K_{M} *(mM)*	k_{cat} *(s*$^{-1}$*)*	$k_{\mathrm{cat}}/K_{\mathrm{M}}$ *(10*^{4} *M*$^{-1}$ *s*$^{-1}$*)*
ATP	0.018 ± 0.001	2.91 ± 0.03	16 ± 1
GTP	0.015 ± 0.002	3.12 ± 0.08	21 ± 3
CTP	0.014 ± 0.002	1.46 ± 0.04	10 ± 1
UTP	0.012 ± 0.001	0.75 ± 0.01	6.2 ± 0.6

Values ± standard error of the parameters of the best fitting hyperbolas for the NS3h NTPase substrate curves shown in Figure S1. Each substrate curve was performed at least three times.
