# Supplementary material for: Steady-State NTPase Activity of Dengue Virus NS3: Number of Catalytic Sites, Nucleotide Specificity and Activation by ssRNA
Source: PLoS One. 2013 Mar 19;8(3):e58508. doi: 10.1371/journal.pone.0058508 (PMC3602377; doi:10.1371/journal.pone.0058508)
Supplement: Text S1 — Deduction of the number of isolated ( iso ), one-side contiguous ( 1s ) and two-side contiguous ( 2s ) sites per base. (PDF) [file pone.0058508.s006.pdf]

Steady-state NTPase activity of dengue virus NS3: number of catalytic sites, nucleotide specificity and activation by ssRNA

J. Jeremías Incicco[1], Leopoldo G. Gebhard[2], Rodolfo M. González-Lebrero[1], Andrea V. Gamarnik[2] and Sergio B. Kaufman[1,*]

1 Instituto de Química y Fisicoquímica Biológicas and Departamento de Química Biológica, Facultad de Farmacia y Bioquímica, Universidad de Buenos Aires, Ciudad Autónoma de Buenos Aires, Argentina.

2 Fundación Instituto Leloir-CONICET, Ciudad Autónoma de Buenos Aires, Argentina.

∗ E-mail: sbkauf@qb.ffyb.uba.ar

Supporting Information Text S1

Deduction of the number of isolated (*iso*), one-side contiguous (*1s*) and two-side contiguous (*2s*) sites per base

Following the reasoning developed in McGhee *et. al.* [1], the average number of free sites per base is factorized as the product between two other average quantities: the number of gaps (composed of free bases between two nearest ligands) per base times the number of free sites per base. That is,

$$< \text{sites per base} > =< \text{gaps per base}>.<\text{sites per gap} > \tag{1}$$

The average number of gaps per base is readily computed from the average number of bound ligands per lattice, $<B>$, divided the average lattice length, N:

$$< \text{gaps per base} >=\frac{< B > +1}{N} = \theta + \frac{1}{N} \approx \theta \tag{2}$$

where θ is the number of bound ligands per base and is given by (see main text):

$$\theta = \frac{[\mathrm{H}_{RC}] + [\mathrm{H}_{RU}] + [\mathrm{HT}_{RC}] + [\mathrm{HT}_{RU}]}{[\text{bases}]_{tot}} \tag{3}$$

The approximation applies as N grows to infinity -hence the limit quality of the resulting equations. Since the number of binding sites in a gap of size g is (g - n + 1), where n is the occluded site size, the average number of sites per gap may be evaluated from the probability distribution of gap sizes, P_g, as:

$$< \text{sites per gap} >= \sum_{g=n}^{N} (g - n + 1).P_g \tag{4}$$

where the lower limit of the sum is n because smaller gaps does not contribute binding sites. To compute P_{g} McGhee *et. al.* [1] made use of the conditional probabilities of finding a base in a free (f) or bound (b) state given that the previous base is in state f or b. Since after one free base it is only possible to find another free base or the first base occupied by a ligand the following relation applies:

$$(ff) + (fb_1) + (fb_2) + \ldots + (fb_n) = (ff) + (fb_1) = 1 \tag{5}$$

where the terms (ij) are conditional probabilities of given a base in state i the following base is in state j; the subindex in b indicates the position within the n bases occluded by each ligand. Similarly, only the last occupied position by a ligand, b_n, may be followed by a free base, whereas other positions within the occluded site are all followed by other occupied bases. Thus, the following relation applies:

$$(b_n f) + (b_n b_1) + (b_n b_2) + \ldots + (b_n b_n) = (b_n f) + (b_n b_1) = 1 \tag{6}$$

These four remainig conditional probabilities are univocally determined through the following relatioships:

$$(1 - n.\theta).(ff) + \theta.(b_n f) = (1 - n.\theta) \tag{7}$$

$$\frac{(ff).(b_n b_1)}{(f b_1).(b_n.f)} = \omega \tag{8}$$

where ω is a cooperativity parameter (see main text). The first equation results from the fact that one base from the fraction of free bases $(1 - n.\theta)$ can be randomly chosen in two mutually exclusive ways: from the right side of a free or of an ocuppied base. The second equation express the relative probability of two bound ligands being in contact or separated by free bases (see McGhee *et. al.* [1]). It is strictly valid under equilibrium conditions but its applicability can be extended to the more general case of *lattice equilibrium* [2], an hypothetical condition in which bound ligands attain the same distribution along the the lattice as that expected for an equilibrium state with the same amount of bound ligands. Therefore, in making use of this equation and the eqautions derived from it, it will be implicit the assumption that the condition of lattice equilibrium is fulfilled during the time courses of ATP hydrolysis from which ATPase activity was obtained.

In terms of the conditional probabilities the distribution of gap sizes can be expressed as:

$$P_g = (b_n f).(ff)^{g-1}.(f b_1) \tag{9}$$

Three kinds of binding sites on the RNA lattice can be distinguished: isolated (*iso*), one-side contiguous (*1s*) and two-side contiguous (*2s*) sites. To compute the number of these sites, it is considered that, within a gap g units sized, the number of isolated sites is two bases smaller than the total number of sites and equals (g - n - 1), whereas the number of one-side contiguous sites is just 2. For the case of two-side contiguous sites it is easily seen that they are only present withing gaps n units sized. Thus we have (cf. equations 14a-c in [1]):

$$< \text{isolated sites per gap} > = \sum_{g=n+2}^{\infty} (g - n - 1).P_g = \frac{(b_n f).(ff)^{n+1}}{(f b_1)} \tag{10}$$

$$< \text{one-side contiguous sites per gap} > = \sum_{g=n+1}^{\infty} 2.P_g = 2.(b_n f).(ff)^n \tag{11}$$

$$< \text{two-side contiguous sites per gap} > = P_n = (b_n f).(ff)^{n-1}(f b_1) \tag{12}$$

The sum of these expressions is simply the average total number of free sites per gap:

$$<\text{sites per gap}> = \sum_{g=n}^{\infty} (g - n + 1).P_g = \frac{(b_n f).(ff)^{n-1}}{(f b_1)} \tag{13}$$

Finally, the average number of sites per base can now be evaluated by substitution of eqs. (13), (14) and (17) in the following expressions:

$$f_{iso}(\theta, n) = <\text{isolated sites per base}> = <\text{gaps per base}>.<<\text{isolated sites per gap}> \tag{14}$$

$$f_{1s}(\theta, n) = <\text{one-side contiguous sites per base}> = <\text{gaps per base}>.<\text{one-side contiguous sites per gap}> \tag{15}$$

$$f_{2s}(\theta, n) = <\text{two-side contiguous sites per base}> = <\text{gaps per base}>.<\text{two-side contiguous sites per gap}> \tag{16}$$

$$f_{tot}(\theta, n) = <\text{sites per base}> = <\text{gaps per base}>.<\text{sites per gap}> \tag{17}$$

We tested three possible definitions of a *crowded* and an *uncrowded* states of NS3 bound to RNA where it is respectively able and unable to catalyze the hydrolysis of ATP: (1) NS3 molecules in single and doubly contiguous sites are in a crowded state; *i.e.*, only NS3 molecules in isolated sites are in the uncrowded state; (2) NS3 molecules in isolated and single contiguous sites are in the uncrowded state; *i.e.*, only NS3 molecules in doubly contiguous sites are in the crowded state; (3) NS3 molecules in one half of single contiguous sites and in doubly contiguous sites are in the crowded state; this corresponds to a model of crowding of a given NS3 molecule on the RNA by the presence of other NS3 molecule in a contiguous base on one of its two sides (see below).

For any of these definitions, the nuber of crowded and uncrowded *binding sites* per base, $f_C(\theta, n)$ and $f_U(\theta, n)$ can be expressed as:

$$f_C(\theta, n) = \omega^2.f_{2s}(\theta, n) + \frac{1}{2}\omega.f_{1s}(\theta, n).s \tag{18}$$

$$f_U(\theta, n) = f_{iso}(\theta, n) + \frac{1}{2}\omega.f_{1s}(\theta, n).(2 - s) \tag{19}$$

where s is the number of sides of a bound NS3h molecule that must be adjacent to a free base in order to be in an uncrowded state. It takes the values 2, 1 and 0, respectively, under the definitions (1), (2) and (3) for the crowded and uncrowded states. The implicit assumption behind equations 25-26 is that $5'$ and $3'$ neighbors occur with equal probabilities and thus contribute equally to the number of one-side contiguous sites.

References

1. McGhee JD, von Hippel PH (1974) Theoretical aspects of DNA-protein interactions: Co-operative and non-co-operative binding of large ligands to a one-dimensional homogeneous lattice. Journal of Molecular Biology 86: 469-489.

2. Epstein IR (1979) Kinetics of nucleic acid-large ligands interactions: exact Monte Carlo treatment and limiting cases of reversible binding. Biopolymers 18: 2037-2050.
